# Variable selection for prediction models of Omicron infection: Insights from two population-based cohort studies

**DOI:** 10.1186/s41512-026-00231-0

**Published:** 2026-05-11

**Authors:** Joanne Lacy, Dominik Menges, Tala Ballouz, Anja Frei, Céline Pellaton, Craig Fenwick, Giuseppe Pantaleo, Milo A. Puhan

**Affiliations:** 1 Epidemiology, Biostatistics and Prevention Institute (EBPI), University of Zurich (UZH), Zurich, Switzerland; 2 Service of Immunology and Allergy, Lausanne University Hospital (CHUV), University of Lausanne (UNIL), Lausanne, Switzerland

## Abstract

**Background:**

During the early stages of the COVID-19 pandemic, prediction modelling was widely used to forecast infection rates while only few studies developed models to predict individual risk of infection for Omicron and subsequent variants. For such prediction models to perform well, it is important to carefully select predictors from a comprehensive set of potential factors, including prior infection and vaccination history, individual behaviors, and immunological markers.

**Methods:**

This exploratory analysis aimed to develop and compare prediction models for Omicron infection to provide an evidence base for identifying key predictors of SARS-CoV-2 infections which can be used to inform future derivation and validation of predictive models. We used data from 710 participants from two ongoing, prospective population-based cohorts: the Zurich SARS-CoV-2 Cohort and the Zurich SARS-CoV-2 Vaccine Cohort (Switzerland). Participants were recruited between 2020 and 2021 and provided demographic data, vaccination history, self-reported infections, and longitudinal serological data (anti-S IgG, anti-S IgA, anti-N IgG antibodies) collected at 6-month intervals. Our main outcome was SARS-CoV-2 infection during the first Omicron wave (01.01.2022–31.03.2022) based on self-reported positive tests or a doubling in any of anti-S IgG, anti-S IgA or anti-N IgG. We used logistic regression models with backward stepwise selection based on the Akaike Information Criterion (AIC) to evaluate predictors and identify the best-fitting models.

**Results:**

Only 17.3% of participants reported a positive SARS-CoV-2 test result during the Omicron wave. However, when including serological testing, 37.2% of participants had evidence of infection, indicating substantial underdiagnosis. The best-performing model had an AUC of 0.69 (95%CI 0.66, 0.73) and included the following predictors: age, sex, compliance with COVID-19 prevention guidelines, smoking status, comorbidities, prior anti-N IgG antibody levels, and the sequence of previous infections and vaccinations. We found that older age (≥ 65 years) was associated with a 50–60% lower odds of Omicron infection across all our models, while having fewer prior exposures (through infections or vaccinations) increased the odds of infection.

**Conclusion:**

This explorative study highlights the importance of integrating comprehensive immunological, clinical and behavioral data to predict SARS-CoV-2 infection risk. Our study lays the foundation to develop and validate future prediction models that identify individuals at risk, particularly through the novel use of infection and prior vaccination sequence as an important predictor.

**Supplementary Information:**

The online version contains supplementary material available at 10.1186/s41512-026-00231-0.

## Background

Prediction modelling on the population level was used during the early stages of the SARS-CoV-2 pandemic for forecasting and to simulate the impact of different interventions such as lockdowns and social distancing measures on infections, hospitalizations and mortality [1]. Such models helped governments plan their responses and mitigation measures of the pandemic [2–6]. Early efforts also included developing diagnostic models to predict the presence or absence of a SARS-CoV-2 infection. For example, the ZOE symptom tracker in the UK used individuals’ symptoms to understand which symptoms were associated with SARS-CoV-2 infection [7, 8]. Many other symptom-based prediction models have been developed to predict infection or presence of antibodies for diagnostic purposes [9–11].

As the pandemic progressed and new immune evading variants emerged, the focus shifted from a population to a more individual level. To tailor vaccination efforts and the use of other protective measures (e.g., masks and social distancing) to individuals, multivariable models to identify individuals at risk increasingly gained interest. One prospective, population-based cohort study in Texas with over 22,000 participants examined factors associated with Omicron infection. They found that ethnicity, household size, vaccine type, and comorbidities were significantly associated with breakthrough infection rates during different waves of the pandemic, with the highest incidence occurring during the Omicron wave [12]. A similar study design found individual predictors of infection among over 300,000 individuals living in Italy who were followed over 3 years [13]. In Switzerland, another study found that individuals with hybrid immunity had a reduced risk of infection during the Omicron period but did not consider infections detected by serology [14].

However, these studies were not primarily designed as clinical prediction models for predicting individual risk. As a consequence, they did not explore a comprehensive set of potential predictors, from demographic factors, first and subsequent infections and vaccinations (and their combinations and sequence), individual behavior or more detailed immunologic predictors. Additionally, unless there is a very extensive population testing, there will always be uncertainty about incident infections in individuals, and results from antibody testing are required in addition to infections diagnosed by PCR or antigen testing. This requires careful consideration of how different sources of information on incident infections are brought together. Thus, before prediction models are developed, a thorough identification of possible predictors and a valid definition of the outcome of infection are required to best inform model derivation and subsequent validation [15]. To the best of our knowledge, no specific predictive models of individual risk for a SARS-CoV-2 infection in the context of the Omicron variant and beyond have yet been developed or validated to date.

A validated prediction model for individual risk for a SARS-CoV-2 infection could inform individuals and health care providers for whom protective behavioral measures and vaccinations are particularly indicated to reduce the risk and severity of infections and would mitigate long-term complications like Long COVID. Therefore, the aims of this study were to (1) develop a definition of incident infections and estimate the proportion of participants from two ongoing prospective, population-based cohort studies who experienced breakthrough infections during the Omicron period; and (2) identify the most relevant predictors of Omicron infections, providing a basis for the development and validation of prediction models to identify individuals at increased risk of infections.

## Methods

The Zurich SARS-CoV-2 Cohort (ZSAC) and Zurich SARS-CoV-2 Vaccine Cohort (ZVAC) are population-based cohorts in the Canton of Zurich, Switzerland. The ZSAC study included 1552 infected individuals who were randomly selected and invited to participate immediately after their first diagnosed SARS-CoV-2 infection between August 2020 to January 2021 (*N* = 1106/1552), or upon study start if infected before August 2020 (*N* = 446/1552) [16, 17]. The ZVAC study enrolled 575 vaccinated individuals who were randomly selected and invited to participate prior to their first vaccination against SARS-CoV-2 between March 2021 and January 2022 (including mRNA vaccines by Moderna and Pfizer/BioNTech and the vector vaccine by Janssen/Johnson & Johnson) [18]. All participants were invited to complete questionnaires at 6-month intervals where they were asked for information on their clinical history, including whether they had had new SARS-CoV-2 infections or vaccines. A subset of prospectively recruited ZSAC participants (*N* = 431/1106) and all ZVAC participants additionally provided repeat blood samples to assess SARS-CoV-2 specific antibodies against the spike (anti-S IgG and anti-S IgA) and nucleocapsid proteins (anti-N IgG), as well as neutralizing antibodies. The assays used for the analysis of anti-S IgA and IgG and anti-N IgG are described in a previous study [19]. In the ZVAC cohort, 13% (75/575) had evidence of prior exposure to SARS-CoV-2 at enrollment based on self-reported infections or positive antibody titers [18].

**Fig. 1:**
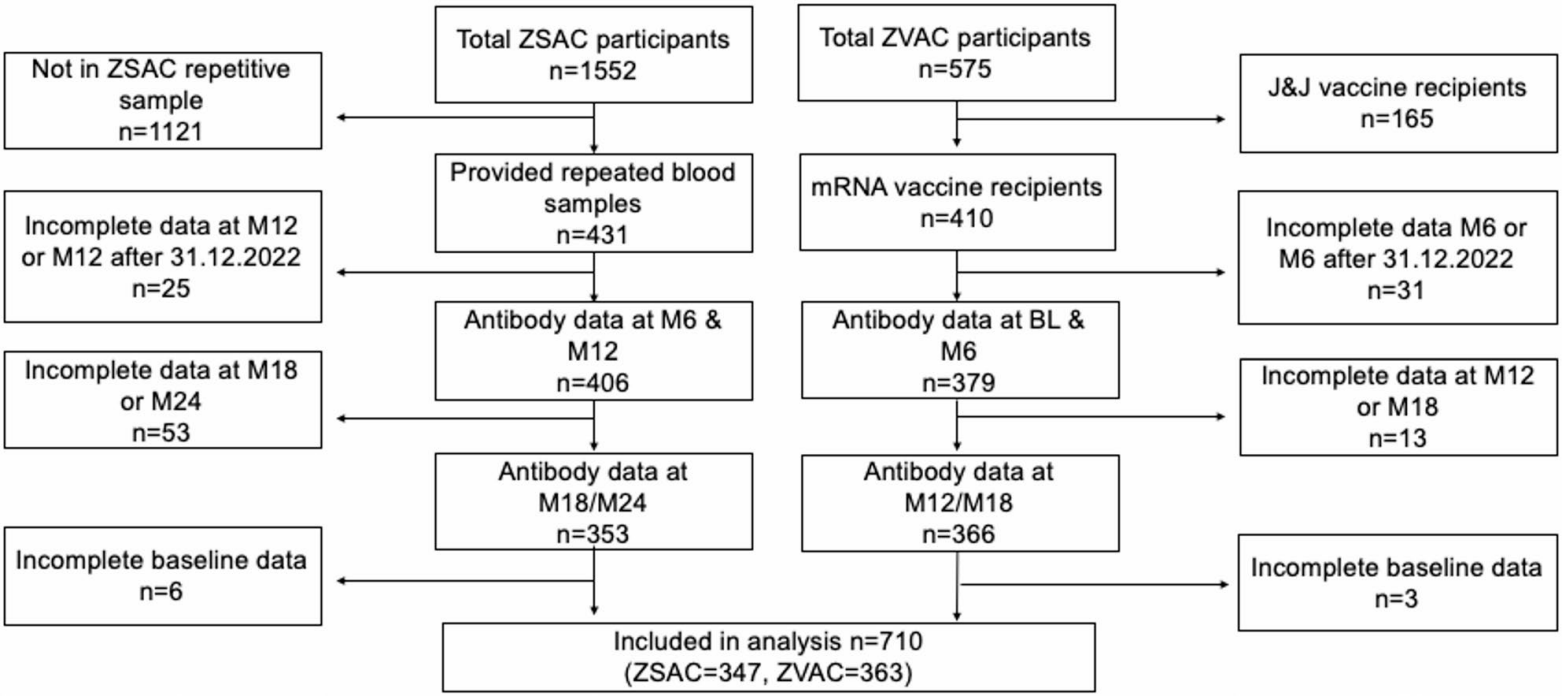
Flowchart of individuals from ZSAC and ZVAC cohort studies into the final analysis

**Fig. 2:**
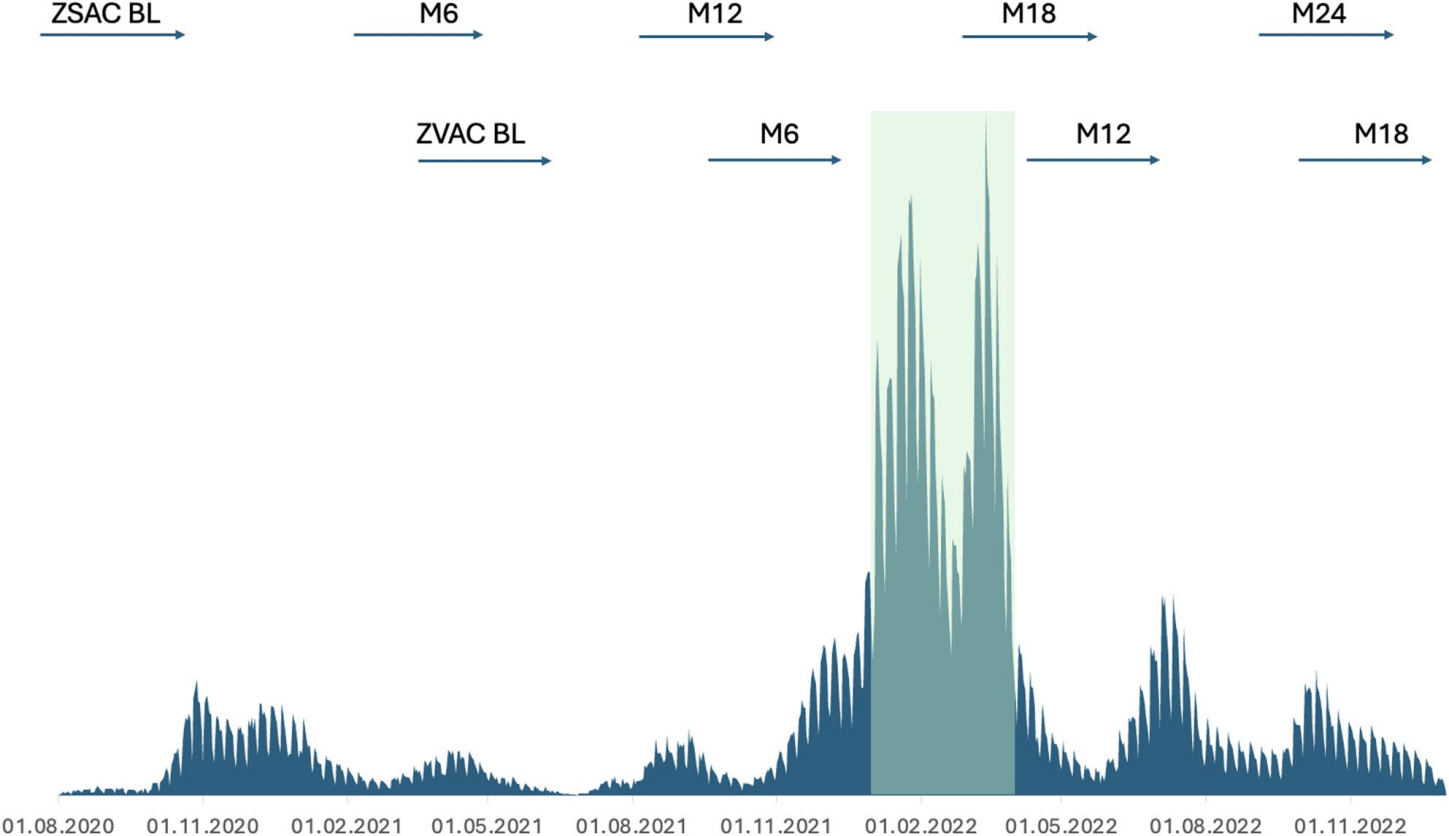
Timeline of ZSAC and ZVAC study visits and background incidence of epidemic in Zürich canton. The arrows representing ZSAC and ZVAC visit dates at each timepoint are used here as a visual guide to the study timelines and are not exactly to scale. Y axis is number of cases diagnosed per day, from 0 to 8000, the highest number of cases was on 14.03.2022 with 7922 cases. Data from: https://www.covid19.admin.ch/en/overview

Our study population for the primary (complete case) analysis consisted of 710 individuals from the ZSAC and ZVAC studies (Fig. 1). We limited our analysis to participants with available repeat blood samples in both studies, and to mRNA-vaccinated individuals in the ZVAC study since those vaccinated with the vector vaccine received their first vaccine in late 2021 and thus did not have a sufficiently long antibody history prior to the start of the Omicron infection period. Our further inclusion criteria were that participants needed to have a study visit at month 6 (M6, ZVAC) or month 12 (M12, ZSAC) with this visit date for each group occurring before 01.01.2022. This meant that they had their blood drawn in autumn 2021, prior to the time window of our outcome definition (see below). They also needed to have a study visit at one or both of their M12/month 18 (M18, ZVAC) timepoints, or their M18/month 24 (M24, ZSAC) timepoints. The study timelines are shown in Fig. 2. Finally, we restricted the analysis to participants with complete baseline demographic and behavioral data. A formal sample size calculation was not performed for this analysis because the sample size was restricted by the number of eligible participants in the underlying cohort studies.

The outcome was a SARS-CoV-2 infection during the first Omicron wave in Switzerland (01.01.2022–31.03.2022), using either self-reported positive SARS-CoV-2 tests (PCR or antigen test) or antibody history to determine infection. For self-reported infections, the reported date of diagnosis was used. An infection based on antibody history was determined if there was a seroconversion (‘negative’ result turning ‘positive’) or a doubling of at least one of anti-S IgG, anti-N IgG or anti-S IgA between two measurement time points. The threshold for interpreting an increase as an infection was based on exploratory analyses evaluating the sensitivity of various outcome definitions in individuals with confirmed infections (Supplementary Fig. 1). An increase in antibodies was counted as an infection if there was an increase in anti-S IgA or IgG in the absence of reported SARS-CoV-2 vaccination, or if there was an increase in anti-N IgG, with antibody levels at the latter time point above the detection threshold. For participants whose infection was determined solely based on antibody history, the most likely date of infection was calculated based on the midpoint between the two study visit dates adjusted for the baseline incidence of SARS-CoV-2 infections in the Canton of Zurich. This adjusted midpoint date was the date at which half of the cumulative number of reported new SARS-CoV-2 cases (a proxy for incidence here) was reached between the two dates. Two sensitivity analyses were performed using alternative outcome definitions: (1) using a more inclusive outcome definition with an extended outcome period (01.01.2022–30.06.2022), and (2) a more pragmatic outcome definition exclusively based on self-reported SARS-CoV-2 infections during the primary time period of interest (01.01.2022–31.03.2022).

In a further exploratory analysis, we categorized participants into clusters with similar longitudinal serological trajectories. For this, we created a cluster variable based on the trajectories of anti-S IgG, anti-S IgA and anti-N IgG antibodies between early and late 2021, corresponding to the baseline and M6 timepoints for ZVAC and M6 and M12 timepoints for ZSAC. We used a k-means clustering method using the *KmL3D* package. Using our previous research [19] and hypothesized trajectories we anticipated 5–8 clusters. We ran the algorithm with 100 runs for each longitudinal *k* cluster and selected the final number of clusters based on the highest Calinski and Harabatz index. The algorithm resulted in 5 clusters with distinct immune trajectories (Supplementary Fig. 2).

We performed logistic regression with the binary outcome of infection based on our pre-specified criteria for infection during the specified time period. Participants who experienced two infections during the specified outcome period were treated the same as those with a single infection, with the date of the first infection considered. The Akaike Information Criteria (AIC) was used to assess which model has the best fit, whereby a decrease of 2 points or more in the AIC was taken as an improvement in model fit [20]. Discrimination was assessed using the area under the receiver operating characteristic curve (AUC), with 95% confidence intervals calculated using DeLong’s method [21]. A sensitivity analysis using multiple imputation by chained equations (MICE) was performed to address missing baseline covariate data, with results based on 20 imputed datasets (*n* = 719; Supplementary Table 3).

We evaluated several models to identify the best predictors related to immune and clinical history for our outcome. Predictors that were included in all models based on prior knowledge [22] included the individuals’ baseline compliance behavior group, age (as a categorical variable), sex, smoking status and a binary variable derived from whether they had any of the following comorbidities: diabetes, cancer, chronic kidney disease, respiratory disease or hypertension. Additional descriptive information on the prevalence of individual comorbidities is provided in Supplementary Table 1. A sensitivity analysis also modelled age as a continuous predictor (per 10-year increase) as an alternative to the primary categorical specification (Supplementary Table 4). Baseline compliance behavior groups were categorized based on the sum of scores from three questions on social distancing, mask wearing and social and hygiene behavior included in the baseline questionnaire (low-medium compliance vs. high compliance with SARS-CoV-2 regulations; Supplementary Fig. 3). The following models were evaluated:

1. Model 1: Included the core set of predictors only —age, sex, smoking behavior, presence of comorbidities, and baseline compliance behavior. From here onwards, this set of predictors will be referred to as “demographic” predictors.
2. Model 2: Included demographic predictors, the time since the last antibody test and anti-N IgG antibodies (measured as log10 transformed mean fluorescence intensity (MFI) ratios) at the last measurement timepoint prior to the start of the outcome period as an indicator of the immune response to prior infections.
3. Model 3: Included demographic predictors, the time since the last antibody test and a predictor for anti-S IgG antibodies (measured as log10 transformed MFI ratios) at the last measurement timepoint prior to the start of the outcome period as an indicator of the combined immune response to prior vaccinations and infections.
4. Model 4: Included demographic predictors, the time since last exposure and a predictor for whether participants had received a booster SARS-CoV-2 vaccination in autumn 2021 (between 01.09.2021 and 31.12.2021) to capture effects of recent vaccination.
5. Model 5: Included demographic predictors, the time since last exposure and a predictor for the type of first exposure (i.e., vaccination or infection) to account for whether there was any effect from the first type of exposure.
6. Model 6: Included demographic predictors, the time since last exposure, a predictors for the total number of previous vaccinations, and a predictor for the total number of previous infections. For the number of vaccinations variable, a primary vaccination course of two mRNA vaccines within 8 weeks of each other was counted as one exposure event.
7. Model 7: Included demographic predictors, the time since last exposure, and a categorical predictor for the sequence of previous vaccinations and infections to take into account both number of exposures and the order of exposure events. After evaluating the exposure sequences across all individuals, only sequences with at least 10 participants were kept as sequences with lower frequencies were too small to provide meaningful analysis. All other sequences were assigned to an “other” category.
8. Model 8: In order to find the model with the best fit, we applied backward stepwise selection using the AIC score as the criterion based on the complete set of predictors to determine the most relevant clinical and immunological predictors to include in the final model. The final model included demographic predictors, the sequence of infections and vaccinations categorical predictor, the time since last exposure, the time since the last antibody test and the anti-N IgG antibodies log10ratio at the last measurement timepoint prior.
9. Model 9: Included demographic predictors, the sequence of infections and vaccinations categorical predictor, the time since last exposure, time since the last antibody test and the anti-S IgG antibodies log10ratio at the last measurement timepoint prior. This post-hoc model was developed by substituting anti-N IgG with anti-S IgG in the best-performing model to assess if this immunological variable, which reflects both infection and vaccination status, improved the predictive properties.
10. Supplementary Immunological trajectory model: Included demographic predictors, time since the last antibody test and a predictor for the immune trajectory clusters based on k-means clustering to assess whether historical antibody patterns (anti-S IgG, anti-N IgG and anti-S IgA) influence the predictions.

Models 4–9 also included a variable for the time since last exposure to take into account the effects of waning immunity from the latest exposure that was included in that model. Models 2–3, models 8–9, and the supplementary immune trajectory model also included a variable for the time since the last antibody test to minimize the bias arising from different time points of blood withdrawal.

All statistical analyses were performed with R version 4.4.1. We used a two-sided alpha of 0.05 as a threshold for statistical significance, without *p*-value adjustment due to the exploratory nature of this study.

## Results

The participants from the two cohort studies had broadly similar characteristics, with 32.0% of participants from ZSAC and 31.1% of participants from ZSAC having at least one comorbidity (Table 1). Participants from ZVAC were slightly older with a median age of 65 compared to 55 in ZSAC and a higher proportion were female (56.5% versus 48.1% in ZSAC) and current smokers (18.5% versus 13.5% in ZSAC). The two cohorts had similarly high levels of baseline compliance behavior with 70.0% of ZSAC participants and 72.5% of ZVAC participants being classified as highly compliant based on their social distancing, masking and hygiene behaviors.

**Table 1:** Baseline demographic data for study population and codified clinical variables

	ZSAC (*N* = 347)	ZVAC (*N* = 363)	Overall (*N* = 710)
Sex			
Female	167 (48.1%)	205 (56.5%)	372 (52.4%)
Male	180 (51.9%)	158 (43.5%)	338 (47.6%)
Age			
18–64 years	210 (60.5%)	169 (46.6%)	379 (53.4%)
65 + years	137 (39.5%)	194 (53.4%)	331 (46.6%)
Median (IQR)	55.0 (38.5 to 68.0)	65.0 (41.5 to 71.0)	59.0 (40.0 to 70.0)
Comorbidity			
No	236 (68.0%)	250 (68.9%)	486 (68.5%)
Yes	111 (32.0%)	113 (31.1%)	224 (31.5%)
Smoking Status			
Non-smoker	213 (61.4%)	206 (56.7%)	419 (59.0%)
Ex-smoker	87 (25.1%)	90 (24.8%)	177 (24.9%)
Smoker	47 (13.5%)	67 (18.5%)	114 (16.1%)
Summary baseline behavior groups^1^			
High compliance	243 (70.0%)	263 (72.5%)	506 (71.3%)
Low-Medium compliance	104 (30.0%)	100 (27.5%)	204 (28.7%)
Anti-N IgG MFI ratio^2^ during autumn 2021			
Mean (SD)	4.5 (5.8)	1.7 (2.9)	3.1 (4.8)
Median [Min, Max]	2.2 [1.0 to 5.3]	1.0 [1.0 to 1.0]	1.0 [1.0 to 3.0]
Anti-S IgG MFI ratio^2^ during autumn 2021			
Mean (SD)	84.0 (39.3)	70.0 (31.8)	76.9 (36.3)
Median [Min, Max]	86.7 [66.3 to 96.4]	69.6 [44.5 to 88.8]	81.2 [50.5 to 94.9]
Anti-S IgA MFI ratio^2^ during autumn 2021			
Mean (SD)	94.3 (142.5)	55.7 (113.9)	74.6 (130.0)
Median [Min, Max]	58.4 [24.6 to 108.3]	22.4 [9.4 to 52.0]	35.5 [13.6 to 80.5]
Days since last antibody test at 01.01.2022			
Mean (SD)	94.7 (30.4	50.5 (28.5)	72.1 (36.8
Median [Min, Max]	99.0 [16.0, 149]	53.0 [3.00, 113]	71.0 [3.00, 149]
Booster dose in autumn 2021			
No	164 (47.3%)	144 (39.7%)	308 (43.4%)
Yes	183 (52.7%)	219 (60.3%)	402 (56.6%)
First type of exposure			
Infection	347 (100.0%)	43 (11.8%)	390 (54.9%)
Vaccination	0 (0.0%)	320 (88.2%)	320 (45.1%)
Total number of vaccinations prior to 01.01.2022			
0	32 (9.2%)	0 (0.0%)	32 (4.5%)
1	142 (40.9%)	143 (39.4%)	285 (40.1%)
2	166 (47.8%)	220 (60.6%)	386 (54.4%)
3	7 (2.0%)	0 (0.0%)	7 (1.0%)
Total number of infections prior to 01.01.2022			
0	0 (0.0%)	310 (85.4%)	310 (43.7%)
1	341 (98.3%)	10 (2.8%)	351 (49.4%)
2	6 (1.7%)	40 (11.0%)	46 (6.5%)
3	0 (0.0%)	3 (0.8%)	3 (0.4%)
Days since last exposure at 01.01.2022			
Mean (SD)	133 (132)	83.0 (83.6)	108 (113)
Median [Min, Max]	98.0 [1.00, 504	36.0 [1.00, 263]	44.5 [1.00, 504]
Sequence of vaccinations and infections			
I	30 (8.6%)	0 (0.0%)	30 (4.2%)
IV	138 (39.8%)	16 (4.4%)	154 (21.7%)
IVV	169 (48.7%)	24 (6.6%)	193 (27.2%)
V	0 (0.0%)	116 (32.0%)	116 (16.3%)
VV	0 (0.0%)	194 (53.4%)	194 (27.3%)
Other	10 (2.9%)	13 (3.6%)	23 (3.2%)
Immune trajectory clusters group^3^			
Group 1	33 (9.5%)	175 (48.2%)	208 (29.3%)
Group 2	26 (7.5%)	149 (41.0%)	175 (24.6%)
Group 3	128 (36.9%)	14 (3.9%)	142 (20.0%)
Group 4	108 (31.1%)	4 (1.1%)	116 (15.8%)
Group 5	52 (15.0%)	21 (5.8%)	73(10.3%)

^1^ Description of compliance behavior groups in Supplementary Fig. 3

^2^ Antibodies samples were read off a Bio-Plex (Luminex) 200 plate reader with Bio-Plex Manager software (version 6.2; Bio-Rad) to obtain a mean fluorescence intensity (MFI). The MFI value for each plasma sample was divided by the mean value of the negative control samples to give an MFI ratio. Seropositivity was determined based on MFI ratio cutoff values exceeding 6.5 for IgA and 6.0 for IgG

^3^Immune trajectory categories are described in Supplementary Fig. 2

There were 123/710 (17.3%) participants that reported a positive SARS-CoV-2 test between 01.01.2022 and 31.03.2022. Out of the 123 individuals with a positive test result, 92.7% (114/123) also had a corresponding seroconversion or increase in anti-N IgG, anti-S IgA, or anti-S IgG according to our definition (Supplementary Fig. 1). Meanwhile, 264/710 (37.2%) of participants had an infection based on our criteria for an infection between 01.01.2022 and 31.03.2022 based on a measured increase in anti-N IgG, anti-S IgA, or anti-S IgG and their adjusted midpoint date of infection. This means that there was an underestimation by 53.4% of SARS-CoV-2 infections that were missed based on diagnosed infections only. A similar underestimation of infections (by 49.1%) was seen for the period between 01.01.2022 and 30.06.2022 where 160/710 (22.5%) of individuals had at least one documented PCR positive infection and 326/710 (45.9%) of individuals had an increase in antibodies indicative of an infection.

We categorized participants into six different vaccine and infection sequence combinations based on their individual clinical trajectories. The biggest categories were one vaccine/a primary vaccine course and then a booster vaccination (VV: 194, 27.3%), an infection and then a vaccine/primary vaccine course as well as a booster vaccination (IVV: 193, 27.2%) and an infection and then one vaccine/primary vaccine course (IV: 154, 21.7%). There were 116 (16.3%) participants with only one vaccine/primary vaccine course and 30 participants (4.2%) had just one infection and no vaccination (Table 1).

Our base model (Model 1 in Table 2) had the fewest number of predictors. This model had an AIC of 909.3 and an AUC of 0.63 (95% CI 0.59, 0.68) and an AIC of 909.3. We added the latest anti-N IgG measurements prior to the Omicron wave to the base model (Model 2) which led to an increase in AIC to 910.3 and AUC to 0.64 (95%CI 0.56, 0.68). Model 3 included anti-S IgG instead of anti-N IgG and had an AIC of 912.8 and an AUC of 0.64 (95%CI 0.60, 0.68).

**Table 2:** Output (Odds Ratios) of multivariable logistic regression models predicting a likely SARS-CoV-2 infection between 01.01.2022 and 31.03.2022

	Infection during January-March 2022 Odds Ratio (95% CI)								
	Model 1	Model 2	Model 3	Model 4	Model 5	Model 6	Model 7	Model 8	Model 9
Prior Anti-N IgG Log10Ratio		0.695 (0.442, 1.079)						0.604 (0.353, 1.017)	
Prior Anti-S IgG Log10Ratio			0.894 (0.517, 1.544)						1.191 (0.625, 2.282)
Days since last antibody test		1.002 (0.998, 1.006)	1.001 (0.997, 1.005)					1.002 (0.997, 1.008)	1.003 (0.998, 1.009)
Booster dose in autumn 2021				1.128 (0.640, 2.023)					
First exposure type: Vaccination					1.077 (0.773, 1.502)				
Total number of SARS-CoV-2 vaccines						1.208 (0.650, 2.278)			
Total number of previous SARS-CoV-2 infection						0.842 (0.642, 1.100)			
Days since last exposure				1.004** (1.001, 1.006)	1.003*** (1.002, 1.005)	1.004* (1.001, 1.008)	1.002 (0.997, 1.007)	1.002 (0.997, 1.006)	1.001 (0.996, 1.006)
Sequence: I							1.348 (0.139, 13.032)	1.700 (0.169, 17.181)	1.721 (0.161, 18.474)
Sequence: IV							1.380 (0.528, 3.594)	1.633 (0.607, 4.399)	1.371 (0.524, 3.587)
Sequence: IVV							1.112 (0.694, 1.783)	1.245 (0.721, 2.146)	0.975 (0.576, 1.645)
Sequence: V							1.751 (0.655, 4.700)	1.964 (0.706, 5.495)	2.105 (0.750, 5.952)
Sequence: Other							0.244* (0.054, 0.786))	0.304 (0.067, 1.001)	0.246* (0.054, 0.795)
Sex: Male	1.023 (0.743, 1.408)	1.020 (0.740, 1.405)	1.018 (0.739, 1.402)	1.015 (0.733, 1.405)	1.012 (0.731, 1.400)	1.017 (0.734, 1.406)	1.051 (0.756, 1.461)	1.037 (0.744, 1.443)	1.045 (0.751, 1.454)
Age group: 65 + years	0.393*** (0.274, 0.560)	0.413*** (0.287, 0.592)	0.397*** (0.276, 0.566)	0.408*** (0.280, 0.589)	0.409*** (0.283, 0.589)	0.393*** (0.269, 0.570)	0.429*** (0.291, 0.627)	0.476*** (0.319, 0.706)	0.436*** (0.295, 0.639)
Comorbidity: Yes	0.904 (0.616, 1.325)	0.872 (0.591, 1.285)	0.889 (0.603, 1.309)	0.939 (0.635, 1.386)	0.953 (0.644, 1.409)	0.950 (0.641, 1.407)	0.917 (0.619, 1.359)	0.879 (0.590, 1.307)	0.902 (0.607, 1.339)
Smoking: Ex-smoker	1.089 (0.741, 1.596)	1.098 (0.746, 1.611)	1.093 (0.743, 1.603)	1.087 (0.736, 1.600)	1.079 (0.731, 1.588)	1.068 (0.723, 1.573)	1.085 (0.731, 1.604)	1.101 (0.741, 1.633)	1.100 (0.740, 1.630)
Smoking: Smoker	1.096 (0.706, 1.692)	1.085 (0.697, 1.680)	1.095 (0.701, 1.701)	1.030 (0.655, 1.608)	1.011 (0.642, 1.580)	1.002 (0.635, 1.570)	0.971 (0.614, 1.524)	0.957 (0.604, 1.505)	0.989 (0.623, 1.557)
Baseline Behaviour Category: Low-medium compliance	1.235 (0.870, 1.748)	1.251 (0.880, 1.774)	1.233 (0.869, 1.747)	1.227 (0.860, 1.746)	1.224 (0.858, 1.742)	1.237 (0.866, 1.762)	1.277 (0.890, 1.827)	1.290 (0.897, 1.850)	1.273 (0.887, 1.824)
AIC	909.3	910.3	912.8	893.4	893.4	893.5	890.5	889.8	893.1
AUC (95% CI De Long)	0.634 (0.592–0.676)	0.641 (0.560–0.682)	0.636 (0.595–0.679)	0.676 (0.636–0.716)	0.674 (0.634–0.715)	0.677 (0.636–0.717)	0.687 (0.648–0.727)	0.694 (0.655–0.733)	0.687 (0.647–0.727)
Observations	710	710	710	710	710	710	710	710	710
Log Likelihood	-447.657	-446.167	-447.393	-437.711	-437.70	-436.765	-432.264	-429.915	-431.573

The table includes Akaike Information Criterion (AIC), and Area Under the Curve (AUC), with 95% confidence intervals for AUC estimated using DeLong’s method. *P* values for each coefficient from the Wald test are denoted by ∗*p* < 0.05*, ∗∗*p* < 0.01, ∗∗∗*p* < 0.001

Models incorporating the participants’ reported previous SARS-CoV-2 vaccinations and infections significantly improved the AIC and the AUC. Model 4, which included a predictor of whether an individual had received an autumn booster, model 5, which incorporated the first type of exposure and model 6, which added the number of previous vaccinations and the number of prior infections to the base model, had similar AICs of 893.4, 893.4 and 893.5, respectively and they all had an AUC of 0.68. When the sequence of vaccinations and infections was added as a predictor to the base model (Model 3), the AIC decreased to 890.5 and the AUC increased to 0.69 (95% CI 0.65, 0.73) suggesting a limited improvement in model fit.

When applying backward stepwise selection to determine the model with the best fit, we found that the model (Model 8) with the lowest AIC of 889.8 and an AUC of 0.69 (95%CI 0.66, 0.73) was a model that included the demographic variables, behavior, anti-N IgG, and sequence of infections and vaccinations. We tried an additional model (Model 9) to assess the predictive performance of a model incorporating Anti-S IgG instead of Anti-N IgG in addition to the sequence and basic demographic variables, which resulted in a model with a similar AUC but a higher AIC of 893.1. A supplementary model adding the immune trajectory clusters based on k-means longitudinal clustering to the base model showed a poorer model fit and lower AIC (917.1) Supplementary Table 2). In a sensitivity analysis using multiple imputation for missing data across all predictors in the final model, effect estimates were similar to those obtained from the complete-case analysis. The imputed analyses increased the sample size from 710 to 719 participants and did not materially change the direction, magnitude, or interpretation of the associations (Supplementary Table 3). When age was modelled as a continuous predictor (per 10-year increase), effect estimates for other predictors were similar to the primary categorical-age model. (Supplementary Table 4).

Individual predictors that remained statistically significant across multiple models included age group, with over 65 years age having 50–60% lower odds of infection with the Omicron variant from 01.03.2022 to 31.03.2022 [21–23]. In terms of the sequence variable, the category with only one infection (I) or infection and subsequent primary vaccination (IV) both had an increased risk of infection when compared to participants with a sequence of primary vaccination + booster vaccination (VV).

In a sensitivity analysis, using reported infections only as the outcome and applying stepwise backwards selection also led to a best performing model that included the same variables as model 8, with an additional predictor being the total number of previous vaccines. This led to very high odds ratios, likely due to data separation, particularly for cases where the sequence is ‘I’ and the total number of vaccines is 0 (Supplementary Table 5). Our expanded outcome period from 01.01.2022–30.06.2022 resulted in a very slightly higher AUC, however the coefficients for the predictors remained consistent with those in the models for our main outcome (Supplementary Table 6).

## Discussion

Over one third of the cohort experienced a SARS-CoV-2 infection based on our infection classification criteria between January and March 2022. This finding is supported by work from the Corona Immunitas group, also based in Switzerland, which found that 34% of participants in their representative sample in March 2022 had serology consistent with infection during this time [23]. The model with the highest predictive performance had an AUC of 0.69 (95%CI 0.66, 0.73) and included the predictors anti-N IgG, age, sex, compliance behavior, smoking behavior, comorbidities and the sequence of infections and vaccinations. Models incorporating additional variables such as anti-S IgG, or those that codified the predictors in other formats—such as specifying whether individuals received an autumn booster or their initial type of exposure—had marginally lower AUC although it should be noted that discrimination alone is not enough to judge model performance in our study, and future model development should also consider calibration, clinical relevance, and validation in independent datasets With the exception of anti-N IgG, the predictors are generally readily available in clinical practice and could be used in a future prediction model.

It is challenging to directly compare our model to others in the literature as they tend to look only at individual predictors and not overall model fit. In terms of individual predictors, a higher anti-N IgG level prior to the Omicron wave appears to be associated with lower odds of infection, with each 10-fold increase in the anti-N IgG MFI ratio being associated with a decrease in the odds of Omicron infection by approximately 39.6% in our best performing model, which is in line with other studies [24, 25]. The effect of imprinting, that is the way the immune system responds to the first exposure, which may affect how it responds to subsequent infections or vaccinations, is not clear from our models and we do not have sufficient evidence to support this proposed theory [26, 27]. However, the sequence of vaccinations and infections gives a useful overview of the different exposures that the participants had. Overall, it appears that fewer previous exposure events (sequence I or V) lead to a higher likelihood of infection compared to individuals with more exposure events. This result is expected as other researchers have found that a booster vaccination or hybrid immunity —where individuals have both recovered from a SARS-CoV-2 infection and been vaccinated, with the term most often referring to those with infection prior to vaccination — is protective against Omicron infections [28].

Including behavior as a predictor also provided some important insights; participants with low-to-moderate baseline compliance with COVID-19 regulations had 23–29% higher odds of contracting an Omicron infection compared to individuals with high baseline compliance, although these results were not statistically significant. This suggests that including behavior in the model is a valuable approach, as it is likely to be a key predictor. However, further exploration is needed, particularly considering the changing COVID-19 guidelines over time and updated questions on compliance from later on in the pandemic would be useful for future analysis. In addition, our finding that older adults have a lower risk of infection with the Omicron variant is in line with other studies, although it is unclear whether this is purely due to behavioral factors such as avoidance of situations with a transmission risk (which were not captured by our behavior variable) or whether there are immunological reasons as well [29–32]. In this study, individual comorbidities were relatively uncommon and comorbidity counts were highly skewed, so we used a binary comorbidity indicator to keep the models parsimonious; this comorbidity predictor was not associated with higher odds of an Omicron infection. Future work could also explore more granular measures of comorbidity, particularly in larger datasets.

The SARS-CoV-2 pandemic has provided an unprecedented opportunity to develop and refine prediction models. The vast amount of data collected during this period offers a foundation that is normally challenging to obtain. While it will be essential to develop and validate these models for other variants and potentially even other coronaviruses, some predictors may have broader applicability, suggesting that their use could extend beyond the current pandemic, contributing to preparedness for future public health challenges. The outcome of our research lays a foundation that can be further developed into a prediction model, particularly using infection and prior vaccination sequence as a predictor. However, based on the data we collected at two previous timepoints, clustering participants algorithmically according to their prior immune response trajectories did not enhance predictive performance. Future research could explore the potential of more detailed longitudinal immune phenotyping with additional timepoints to offer additional insights. Our approach has not been widely explored before, and it will be crucial to confirm our findings in diverse populations with varying exposure histories. New cohorts or existing cohorts could be used to develop a statistical model using the predictors we have found to create a clinical prediction model in line with the standard process [33]. The model would then have to be validated to test its performance across different populations, involving both internal and external validation processes. We used an AIC-based approach to compare pre-specified predictors and to select an interpretable model. Penalised regression methods, such as the lasso or elastic net, could be an alternative strategy for variable selection and may have advantages in settings with correlated predictors or higher-dimensional feature spaces and this is something that could also be explored with future studies.

Our study also has some limitations. First, this study represents an exploratory secondary analysis of two existing cohorts with a fixed sample size and without prospective registration of this specific analysis; accordingly, the modelling approach was developed within the constraints of the available data. Second, infection status was defined using self-reported data and serological criteria rather than clinical adjudication, which may have resulted in misclassification and the practicalities of testing individuals for anti-S IgG, anti-S IgA and anti-N IgG before and after infection is something that needs to be considered. When establishing prediction models and diagnosed infections are likely to be incomplete, an alternative outcome definition may need to be used. This may include a symptom-based outcome (e.g., flu-like illness) instead of antibody testing in digital cohort studies. The reliability of participants’ ability to recall previous infections could be challenging in cohorts without digital health records or in a situation without readily available SARS-CoV-2 testing. We also did not explore the severity of infections which represents an important direction for future research, particularly in relation to healthcare burden and prioritisation of protective measures. However, our sensitivity analysis using only diagnosed cases may indicate more severe infections compared to those identified solely through antibody increases. Although missingness was minimal and results were stable in multiple-imputation sensitivity analyses, some bias may remain if missingness was not completely at random.

Third, participants had study visits for blood sampling at different times in the calendar year depending on which study they were enrolled in (ZSAC or ZVAC). As a result, there may be a systematic bias related to the exposure and consequently infection across the two cohorts, which may impact predictive properties of variables highly associated with study membership (e.g. type of first exposure or sequence). However, we mitigated this bias by using either reported or expected dates of infection that were adjusted based on the underlying SARS-CoV-2 incidence in Zurich. Furthermore, sensitivity analyses using an extended outcome period from 01.01.2022–30.06.2022 (thus including a different weighting of the time exposed in the two cohorts with respect to the peak incidence of Omicron infections) resulted in models that were broadly consistent with our main analysis using a shorter timeframe (01.01.2022–31.03.2022). Based on this, we are confident that the slightly different timelines of the studies do not introduce a relevant bias with respect to our findings.

## Conclusion

Overall, the predictors that performed well in this setting include readily available data such as the sequence of previous infections and vaccinations. The applicability of this research to develop and validate prediction models for SARS-CoV-2 infections including other variants and in other settings requires further evaluation.

## Supplementary Information

Supplementary Material 1.

## Data Availability

The data is available from the corresponding author (miloalan.puhan@uzh.ch) upon reasonable request.

## References

[1] Wynants L, Van Calster B, Collins GS, Riley RD, Heinze G, Schuit E et al. Prediction models for diagnosis and prognosis of covid-19: systematic review and critical appraisal. BMJ. 2020;369:29. Available from: https://www.bmj.com/content/369/bmj.m1328. Cited 29 2024. 10.1136/bmj.m1328 PMC7222643 32265220

[2] Yan L, Zhang HT, Goncalves J, Xiao Y, Wang M, Guo Y et al. An interpretable mortality prediction model for COVID-19 patients. Nature Machine Intelligence 2020 2:5. 2020;2(5):283–8. Available from: https://www.nature.com/articles/s42256-020-0180-7. Cited 13 Aug 2024.

[3] Zhao Z, Chen A, Hou W, Graham JM, Li H, Richman PS et al. Prediction model and risk scores of ICU admission and mortality in COVID-19. PLoS One. 2020;15(7):e0236618. Available from: https://journals.plos.org/plosone/article?id=10.1371/journal.pone.0236618. Cited 13 Aug 2024. 10.1371/journal.pone.0236618 PMC7392248 32730358

[4] Jehi L, Ji X, Milinovich A, Erzurum S, Rubin BP, Gordon S, et al. Individualizing Risk Prediction for Positive Coronavirus Disease 2019 Testing: Results From 11,672 Patients. Chest. 2020;158(4):1364–75. 32533957 10.1016/j.chest.2020.05.580 PMC7286244

[5] Gong J, Ou J, Qiu X, Jie Y, Chen Y, Yuan L et al. A Tool for Early Prediction of Severe Coronavirus Disease 2019 (COVID-19): A Multicenter Study Using the Risk Nomogram in Wuhan and Guangdong, China. Clinical Infectious Diseases. 2020;71(15):833–40. Available from: 10.1093/cid/ciaa443. Cited 13 Aug 2024. 10.1093/cid/ciaa443 PMC7184338 32296824

[6] Hoertel N, Blachier M, Blanco C, Olfson M, Massetti M, Rico MS et al. A stochastic agent-based model of the SARS-CoV-2 epidemic in France. Nature Medicine 2020 26:9. 2020;26(9):1417–21. Available from: https://www.nature.com/articles/s41591-020-1001-6. Cited 13 Aug 2024. 10.1038/s41591-020-1001-6 32665655

[7] Menni C, Valdes AM, Freidin MB, Sudre CH, Nguyen LH, Drew DA et al. Real-time tracking of self-reported symptoms to predict potential COVID-19. Nature Medicine 2020 26:7. 2020;26(7):1037–40. Available from: https://www.nature.com/articles/s41591-020-0916-2. Cited 14 Aug 2024. 10.1038/s41591-020-0916-2 PMC7751267 32393804

[8] Drew DA, Nguyen LH, Steves CJ, Menni C, Freydin M, Varsavsky T et al. Rapid implementation of mobile technology for real-time epidemiology of COVID-19. Science. 2020;368(6497):1362–7. Available from: https://pubmed.ncbi.nlm.nih.gov/32371477/. Cited 14 Aug 2024. 10.1126/science.abc0473 PMC7200009 32371477

[9] Ramírez Varela A, Moreno López S, Contreras-Arrieta S, Tamayo-Cabeza G, Restrepo-Restrepo S, Sarmiento-Barbieri I, et al. Prediction of SARS-CoV-2 infection with a Symptoms-Based model to aid public health decision making in Latin America and other low and middle income settings. Prev Med Rep. 2022;27:101798. 35469291 10.1016/j.pmedr.2022.101798 PMC9020649

[10] Lehmann J, Giesinger JM, Rumpold G, Borena W, Knabl L, Falkensammer B et al. Estimating seroprevalence of SARS-CoV-2 antibodies using three self-reported symptoms: development of a prediction model based on data from Ischgl, Austria. Epidemiol Infect. 2021;149:e52. Available from: https://www.cambridge.org/core/journals/epidemiology-and-infection/article/estimating-seroprevalence-of-sarscov2-antibodies-using-three-selfreported-symptoms-development-of-a-prediction-model-based-on-data-from-ischgl-austria/734CF75E4555B1F730350EDCD7D4D9F7. Cited 28 Aug 2024. 10.1017/S0950268821000418 PMC7925980 33597049

[11] Zoabi Y, Deri-Rozov S, Shomron N. Machine learning-based prediction of COVID-19 diagnosis based on symptoms. npj Digital Medicine 2021 4:1. 2021;4(1):1–5. Available from: https://www.nature.com/articles/s41746-020-00372-6. Cited 28 Aug 2024. 10.1038/s41746-020-00372-6 PMC7782717 33398013

[12] Desantis SM, Yaseen A, Hao T, León-Novelo L, Talebi Y, Valerio-Shewmaker MA et al. Incidence and Predictors of Breakthrough and Severe Breakthrough Infections of SARS-CoV-2 After Primary Series Vaccination in Adults: A Population-Based Survey of 22 575 Participants. J Infect Dis. 2023;227(10):1164–72. Available from: 10.1093/infdis/jiad020. Cited 28 Aug 2024. 10.1093/infdis/jiad020 36729177

[13] Flacco ME, Acuti Martellucci C, Soldato G, Di Martino G, Rosso A, Carota R et al. Predictors of SARS-CoV-2 Infection and Severe and Lethal COVID-19 after Three Years of Follow-Up: A Population-Wide Study. Viruses. 2023;15(9):1794. Available from: https://www.mdpi.com/1999-4915/15/9/1794/htm. Cited 28 Aug 2024. 10.3390/v15091794 PMC10534678 37766201

[14] B BF SG et al. T E, O L, A B, E L,. Risk and symptoms of COVID-19 in health professionals according to baseline immune status and booster vaccination during the Delta and Omicron waves in Switzerland-A multicentre cohort study. PLoS Med. 2022;19(11). Available from: https://pubmed.ncbi.nlm.nih.gov/36342956/. Cited 21 Mar 2024. 10.1371/journal.pmed.1004125 PMC9678290 36342956

[15] Steyerberg EW, Moons KGM, van der Windt DA, Hayden JA, Perel P, Schroter S et al. Prognosis Research Strategy (PROGRESS) 3: Prognostic Model Research. PLoS Med. 2013;10(2):e1001381. Available from: https://journals.plos.org/plosmedicine/article?id=10.1371/journal.pmed.1001381. Cited 24 Sep 2024. 10.1371/journal.pmed.1001381 PMC3564751 23393430

[16] Ballouz T, Menges D, Anagnostopoulos A, Domenghino A, Aschmann HE, Frei A et al. Recovery and symptom trajectories up to two years after SARS-CoV-2 infection: population based, longitudinal cohort study. BMJ. 2023;381. Available from: https://www.bmj.com/content/381/bmj-2022-074425. Cited 12 Nov 2024. 10.1136/bmj-2022-074425 PMC10230608 37257891

[17] Menges D, Ballouz T, Anagnostopoulos A, Aschmann HE, Domenghino A, Fehr JS et al. Burden of post-COVID-19 syndrome and implications for healthcare service planning: A population-based cohort study. PLoS One. 2021;16(7):e0254523. Available from: https://journals.plos.org/plosone/article?id=10.1371/journal.pone.0254523. Cited 12 Nov 2024. 10.1371/journal.pone.0254523 PMC8274847 34252157

[18] Bürzle O, Menges D, Maier JD, Schams D, Puhan MA, Fehr J et al. Adverse effects, perceptions and attitudes related to BNT162b2, mRNA-1273 or JNJ-78436735 SARS-CoV-2 vaccines: Population-based cohort. npj Vaccines 2023 8:1. 2023;8(1):1–10. Available from: https://www.nature.com/articles/s41541-023-00657-3. Cited 12 Nov 2024. 10.1038/s41541-023-00657-3 PMC10123463 37095137

[19] Menges D, Zens KD, Ballouz T, Caduff N, Llanas-Cornejo D, Aschmann HE et al. Heterogenous humoral and cellular immune responses with distinct trajectories post-SARS-CoV-2 infection in a population-based cohort. Nature Communications 2022 13:1. 2022;13(1):1–16. Available from: https://www.nature.com/articles/s41467-022-32573-w. Cited 10 May 2024. 10.1038/s41467-022-32573-w PMC9386650 35982045

[20] Cavanaugh JE, Neath AA. The Akaike information criterion: Background, derivation, properties, application, interpretation, and refinements. Wiley Interdiscip Rev Comput Stat. 2019;11(3):e1460. Available from: https://onlinelibrary.wiley.com/doi/full/10.1002/wics.1460. Cited 14 Nov 2024.

[21] DeLong ER, DeLong DM, Clarke-Pearson DL. Comparing the areas under two or more correlated receiver operating characteristic curves: a nonparametric approach. Biometrics. 1988;44(3):837–45. Available from: https://pubmed.ncbi.nlm.nih.gov/3203132/. Cited 23 Jan 2026. 3203132

[22] Harris M, Hart J, Bhattacharya O, Russell FM. Risk factors for SARS-CoV-2 infection during the early stages of the COVID-19 pandemic: a systematic literature review. Front Public Health. 2023;11:1178167. 37583888 10.3389/fpubh.2023.1178167 PMC10424847

[23] Amati R, Frei A, Kaufmann M, Sabatini S, Pellaton C, Fehr J et al. Functional immunity against SARS-CoV-2 in the general population after a booster campaign and the Delta and Omicron waves, Switzerland, March 2022. Eurosurveillance. 2022;27(31):2200561. Available from: https://www.eurosurveillance.org/content/10.2807/1560-7917.ES.2022.27.31.2200561. Cited 14 Aug 2024. 10.2807/1560-7917.ES.2022.27.31.2200561 PMC9358404 35929427

[24] Cohen D, Izak M, Stoyanov E, Mandelboim M, Perlman S, Amir Y et al. Predictors of reinfection with pre-Omicron and Omicron variants of concern among individuals who recovered from COVID-19 in the first year of the pandemic. Int J Infect Dis. 2023;132:72–9. Available from: https://pubmed.ncbi.nlm.nih.gov/37072052/. Cited 19 Nov 2024. 10.1016/j.ijid.2023.04.395 PMC10106114 37072052

[25] Dowell AC, Waiblinger D, Wright J, Ladhani SN, Moss P. Nucleocapsid-specific antibodies as a correlate of protection against SARS-CoV-2 reinfection in children. J Infect. 2023;87(3):267–9. Available from: https://pubmed.ncbi.nlm.nih.gov/37391077/. Cited 19 Nov 2024. 10.1016/j.jinf.2023.06.018 PMC10303317 37391077

[26] Rodda LB, Morawski PA, Pruner KB, Fahning ML, Howard CA, Franko N et al. Imprinted SARS-CoV-2-specific memory lymphocytes define hybrid immunity. Cell. 2022;185(9):1588–1601.e14. Available from: http://www.cell.com/article/S0092867422003282/fulltext. Cited 14 Sep 2023. 10.1016/j.cell.2022.03.018 PMC8926873 35413241

[27] Park YJ, Pinto D, Walls AC, Liu Z, De Marco A, Benigni F et al. Imprinted antibody responses against SARS-CoV-2 Omicron sublineages. Science. 2022;378(6620):619–27. Available from: https://pubmed.ncbi.nlm.nih.gov/36264829/. Cited 14 Sep 2023. 10.1126/science.adc9127 PMC12945441 36264829

[28] Bobrovitz N, Ware H, Ma X, Li Z, Hosseini R, Cao C et al. Protective effectiveness of previous SARS-CoV-2 infection and hybrid immunity against the omicron variant and severe disease: a systematic review and meta-regression. Lancet Infect Dis. 2023;23(5):556–67. Available from: http://www.thelancet.com/article/S1473309922008015/fulltext. Cited 28 Aug 2024. 10.1016/S1473-3099(22)00801-5 PMC10014083 36681084

[29] Gilboa M, Gonen T, Barda N, Cohn S, Indenbaum V, Weiss-Ottolenghi Y et al. Factors Associated With Protection From SARS-CoV-2 Omicron Variant Infection and Disease Among Vaccinated Health Care Workers in Israel. JAMA Netw Open. 2023;6(5):e2314757–e2314757. Available from: https://jamanetwork.com/journals/jamanetworkopen/fullarticle/2805172. Cited 19 Nov 2024. 10.1001/jamanetworkopen.2023.14757 PMC10208153 37219906

[30] Echevarria FM, Caputo M, Camp D, Reddy S, Achenbach CJ. Incidence and risk factors of SARS-CoV-2 breakthrough infection in the early Omicron variant era among vaccinated and boosted individuals in Chicago. PLoS One. 2024;19(8):e0302338. Available from: https://pmc.ncbi.nlm.nih.gov/articles/PMC11299831/. Cited 19 Nov 2024. 10.1371/journal.pone.0302338 PMC11299831 39102410

[31] Walmsley S, Nabipoor M, Lovblom LE, Ravindran R, Colwill K, McGeer A et al. Predictors of Breakthrough SARS-CoV-2 Infection after Vaccination. Vaccines. 2024, Vol 12, Page 36. 2023;12(1):36. Available from: https://www.mdpi.com/2076-393X/12/1/36/htm. Cited 19 Nov 2024. 10.3390/vaccines12010036 PMC10820583 38250849

[32] Reynolds CJ, Pade C, Gibbons JM, Otter AD, Lin KM, Sandoval DM et al. Immune boosting by B.1.1.529 (Omicron) depends on previous SARS-CoV-2 exposure. Science. 2022;377(6603). Available from: https://pubmed.ncbi.nlm.nih.gov/35699621/. Cited 14 Sep 2023. 10.1126/science.abq1841 PMC9210451 35699621

[33] Efthimiou O, Seo M, Chalkou K, Debray T, Egger M, Salanti G. Developing clinical prediction models: a step-by-step guide. BMJ. 2024;386:e078276. Available from: https://www.bmj.com/content/386/bmj-2023-078276. Cited 19 Nov 2024. 10.1136/bmj-2023-078276 PMC11369751 39227063

