# Supplementary material for: Variable selection for prediction models of Omicron infection: Insights from two population-based cohort studies
Source: Diagn Progn Res. 2026 May 11;10:14. doi: 10.1186/s41512-026-00231-0 (PMC13159202; doi:10.1186/s41512-026-00231-0)

**
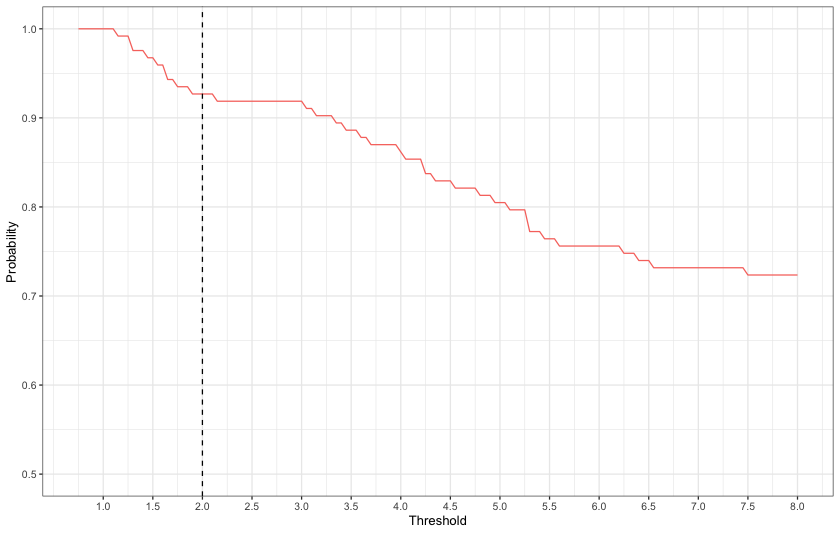
**

**Supplementary Figure 1:**

Figure showing the probability that a participant with a confirmed positive SARS-CoV-2 test result between 01.01.2022-31.03.2022 also meets the case defintion of an infection detected by different thresholds of increases in any of anti-S IgG, anti-S IgA and anti-N IgG (N=123

). The line does not reach zero as participants with a seroconversion were also counted as a case, independent of the selected threshold. A threshold of a two-fold increase was chosen for this study, corresponding to a 92.7% probability of meeting the case definition based on antibodies among those with a diagnosed infection.


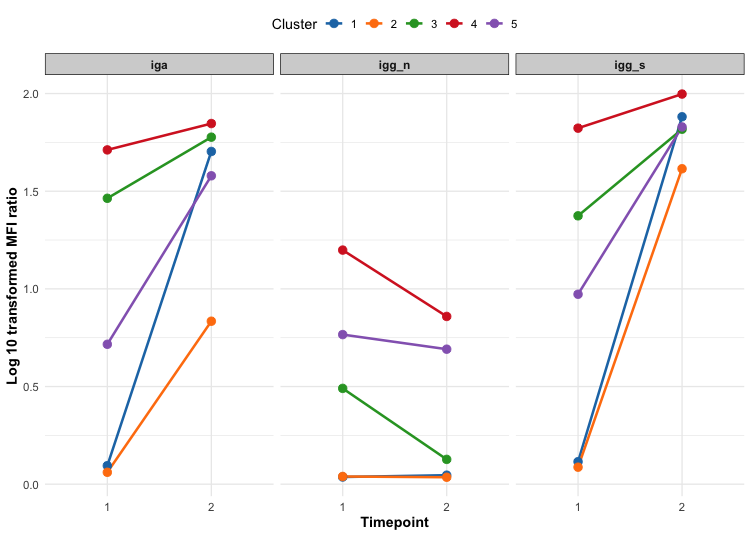


**Supplementary Figure 2:** Visualization of the Mean Log_10_ MFI Ratios of Anti-S IgG, Anti-N IgG and Anti-S IgA for the five clusters identified from longitudinal K-Means Clustering. Timepoint 1 refers to Baseline for ZVAC and Month 6 for ZSAC, timepoint 2 refers to Month 6 for ZVAC and Month 12 for ZSAC.

After applying the clustering algorithm, 1 individual from ZSAC with missing data at month 6 was assigned to group 3 which was the most common immune trajectory group for individuals with the same sequence (IVV) after the clustering method did not assign them to a cluster.


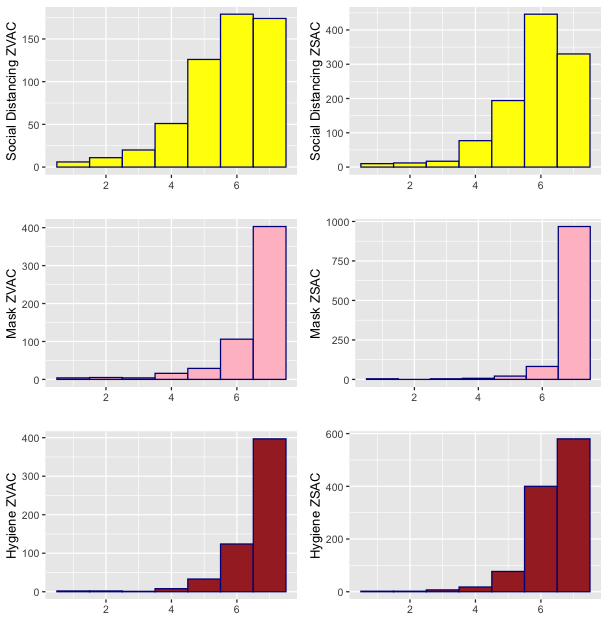


**Supplementary Figure 3:**
Histogram of the behavior scores from the ZSAC and ZVAC questionnaires after imputation for the three variables used to create the summary behaviour variable. These variables are Social Distancing which measured how well they adhered to social distancing measures, Mask which measured how well they adhered to mandatory mask wearing and Hygiene which measured how well they adhered to hygiene recommendations.

Behaviour summary scores were summed from these three variables from the baseline questionnaires for ZSAC and ZVAC participants. These three questions were scored on a scale of 1 to 7, with 7 being “always” (high compliance) and 1 being “never” (low compliance). There was an option for no response, if individuals did not respond or choose the option to not respond then their values were transformed into NA values. When an individual had NA values for all three behavior questions, then they were excluded from the dataset. If they answered 1 or 2 of the questions, the value of the missing variables was imputed from the mean value of their individual response to the other behaviour variables. A categorical variable was created with participants with a total score above 18 having “high compliance” and participants with a total score of 18 or under categorized as “low to medium compliance”.

The English translations of the questions from the baseline ZSAC questionnaire are as follows:

To what extent would you say that it was generally possible for you in the last two weeks before this questionnaire to:

1. To implement the recommended measures for "social distancing" (adhere to the minimum distance of 1.5 meters, no shaking hands or hugs, etc.)
2. In the places where it is currently mandatory to wear a mask (e.g. in public transport)
3. To implement the recommended hygiene measures (regular hand washing, sneezing in the elbows, use of handkerchiefs, etc.)

The English translations of the questions from the baseline ZVAC questionnaire are as follows:

To what extent would you say that it was possible for you last month to:

1. To implement the recommended measures for "social distancing" (adhere to the minimum distance of 1.5 meters, no shaking hands or hugs, etc.)
2. To wear a mask outside of home (if a distance of 1.5 meters was not possible and in places where it is mandatory)

To implement the recommended hygiene measures (regular hand washing, sneezing in the elbows, use of handkerchiefs, etc.)

**Supplementary Table 1:** Prevalence of individual comorbidities included in the binary comorbidity indicator.

|  | **ZSAC (N=347)** | **ZVAC (N=363)** | **Overall (N=710)** |
| --- | --- | --- | --- |
| **Comorbidity (binary)** |  |  |  |
| No | 236 (68.0%) | 250 (68.9%) | 486 (68.5%) |
| Yes | 111 (32.0%) | 113 (31.1%) | 224 (31.5%) |
| **Comorbidity count** |  |  |  |
| Mean (SD) | 0.4 (0.7) | 0.4 (0.8) | 0.4 (0.7) |
| Median (IQR) | 0.0 (0.0 to 1.0) | 0.0 (0.0 to 1.0) | 0.0 (0.0 to 1.0) |
| Range | 0 to 4 | 0 to 4 | 0 to 4 |
| **Hpertension** |  |  |  |
| No | 287 (83.2%) | 291 (80.4%) | 578 (81.8%) |
| Yes | 58 (16.8%) | 71 (19.6%) | 129 (18.2%) |
| Missing | 2 (0.6%) | 1 (0.3%) | 3 (0.4%) |
| **Diabetes** |  |  |  |
| No | 327 (97.3%) | 340 (96.6%) | 667 (96.9%) |
| Yes | 9 (2.7%) | 12 (3.4%) | 21 (3.1%) |
| Missing | 11 (3.2%) | 11 (3.0%) | 22 (3.1%) |
| **CVD** |  |  |  |
| No | 318 (93.3%) | 330 (93.2%) | 648 (93.2%) |
| Yes | 23 (6.7%) | 24 (6.8%) | 47 (6.8%) |
| Missing | 6 (1.7%) | 9 (2.5%) | 15 (2.1%) |
| **Respiratory** |  |  |  |
| No | 313 (92.3%) | 331 (93.5%) | 644 (92.9%) |
| Yes | 26 (7.7%) | 23 (6.5%) | 49 (7.1%) |
| Missing | 8 (2.3%) | 9 (2.5%) | 17 (2.4%) |
| **CKD** |  |  |  |
| No | 338 (99.4%) | 354 (99.4%) | 692 (99.4%) |
| Yes | 2 (0.6%) | 2 (0.6%) | 4 (0.6%) |
| Missing | 7 (2.0%) | 7 (1.9%) | 14 (2.0%) |
| **Cancer** |  |  |  |
| No | 321 (94.1%) | 331 (93.0%) | 652 (93.5%) |
| Yes | 20 (5.9%) | 25 (7.0%) | 45 (6.5%) |
| Missing | 6 (1.7%) | 7 (1.9%) | 13 (1.8%) |

**Supplementary table 2:** Output (Odds Ratios) of a multivariable logistic regression model predicting a likely SARS-CoV-2 infection between 01.01.2022 and 31.03.2022 using the k-means clustering derived immune trajectories variable . The table includes Akaike Information Criterion (AIC), and Area Under the Curve (AUC). P values for each coefficient from the Wald test are denoted by ∗p<0.05* , ∗∗p<0.01, ∗∗∗p<0.001


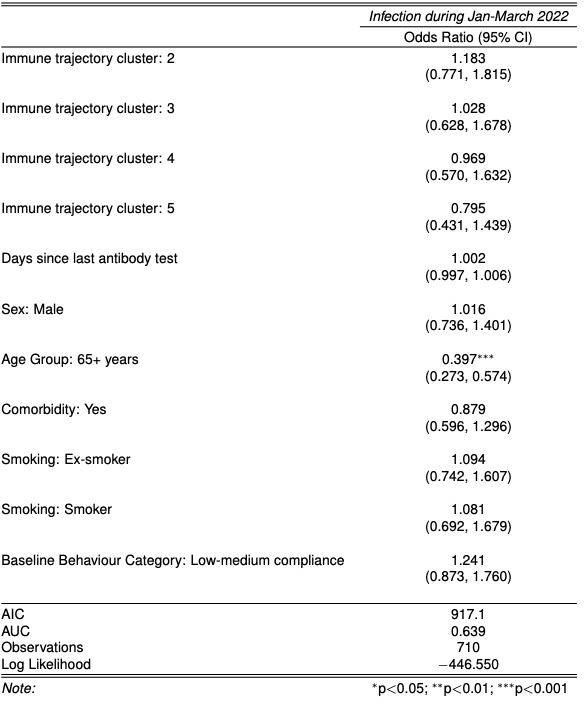


**Supplementary Table 3:** Odds ratios with 95% confidence intervals from the complete-case analysis (n = 710) and pooled multiple-imputation analysis (n = 719). Multiple imputation was performed for all predictors included in the final model (model 8) with estimates pooled across 20 imputations using Rubin’s rules.

|  | **OR estimate Complete case** | **OR estimate Multiple imputation** | **conf.low Complete case** | **conf.low Multiple imputation** | **Conf high Complete case** | **Conf high Multiple imputation** |  | **p.value Multiple imputation** |
| --- | --- | --- | --- | --- | --- | --- | --- | --- |
| **Predictor** |  |  |  |  |  |  | **p.value Complete case** |  |
| igg_n_log10ratio | 0.6 | 0.644 | 0.351 | 0.393 | 1.01 | 1.056 | 0.058 | 0.081 |
| days since last ab test | 1.002 | 1.002 | 0.997 | 0.997 | 1.008 | 1.007 | 0.433 | 0.427 |
| days since last exposure | 1.002 | 0.999 | 0.997 | 0.994 | 1.006 | 1.003 | 0.526 | 0.646 |
| sequence_catI | 1.738 | 1.452 | 0.173 | 0.166 | 17.59 | 12.736 | 0.638 | 0.736 |
| sequence_catIV | 1.661 | 1.84 | 0.618 | 0.728 | 4.479 | 4.653 | 0.314 | 0.197 |
| sequence_catIVV | 1.269 | 1.31 | 0.736 | 0.777 | 2.188 | 2.209 | 0.391 | 0.311 |
| sequence_catOther | 0.308 | 0.3 | 0.068 | 0.083 | 1.014 | 1.085 | 0.078 | 0.066 |
| sequence_catV | 1.978 | 2.588 | 0.71 | 0.999 | 5.538 | 6.706 | 0.192 | 0.05 |
| sexmale | 1.036 | 1.059 | 0.744 | 0.782 | 1.442 | 1.434 | 0.835 | 0.711 |
| age_group65+ years | 0.478 | 0.58 | 0.32 | 0.397 | 0.71 | 0.847 | 0 | 0.005 |
| comorbidityYes | 0.879 | 0.861 | 0.59 | 0.594 | 1.307 | 1.249 | 0.523 | 0.43 |
| smokingEx-smoker | 1.102 | 1.081 | 0.741 | 0.749 | 1.633 | 1.561 | 0.63 | 0.676 |
| smokingSmoker | 0.961 | 1.003 | 0.606 | 0.66 | 1.511 | 1.525 | 0.863 | 0.987 |
| behaviour_category: low-medium | 1.293 | 1.123 | 0.903 | 0.809 | 1.848 | 1.558 | 0.159 | 0.489 |

**Supplementary Table 4:** Odds ratios with 95% confidence intervals from the final model (model 8) with age specified as a continuous predictor (per 10-year increase), compared with the primary analysis using categorical age (age groups of 18-64 and 65+).

|  | **OR_binary** | **OR_cont** | **lowCI_binary** | **lowCI_cont** | **highCI_binary** | **highCI_cont** |
| --- | --- | --- | --- | --- | --- | --- |
|  |  |  |  |  |  |  |
| igg_n_log10ratio | 0.599876 | 0.613002 | 0.351152 | 0.358692 | 1.010418 | 1.0333954 |
| days since last ab test | 1.002152 | 1.00246 | 0.996788 | 0.997076 | 1.007574 | 1.007902 |
| days since last exposure | 1.001579 | 1.002088 | 0.996681 | 0.997157 | 1.006483 | 1.0070212 |
| sequence_catI | 1.738483 | 1.344973 | 0.17271 | 0.13249 | 17.5936 | 13.7658513 |
| sequence_catIV | 1.661218 | 1.435917 | 0.617516 | 0.528325 | 4.478789 | 3.9105407 |
| sequence_catIVV | 1.269226 | 1.222098 | 0.73553 | 0.709222 | 2.18762 | 2.1050069 |
| sequence_catOther | 0.307979 | 0.27506 | 0.067603 | 0.05981 | 1.014009 | 0.9162299 |
| sequence_catV | 1.977573 | 1.735433 | 0.709714 | 0.617443 | 5.537851 | 4.9011726 |
| sexmale | 1.035789 | 1.059332 | 0.743728 | 0.759529 | 1.441672 | 1.476925 |
| comorbidityYes | 0.878693 | 0.938358 | 0.590343 | 0.627513 | 1.30695 | 1.4020072 |
| smokingEx-smoker | 1.101891 | 1.201443 | 0.740951 | 0.804468 | 1.633445 | 1.7897678 |
| smokingSmoker | 0.960753 | 0.981084 | 0.606439 | 0.617507 | 1.511348 | 1.5476216 |
| behaviour_category: low-medium | 1.29294 | 1.196146 | 0.90322 | 0.828737 | 1.847666 | 1.7213149 |
| **age (10y interval continous vs 65+ for categorical)** | **0.478** | **0.783** | **0.32** | **0.696** | **0.71** | **0.878** |

**Supplementary Table 5:** Output (Odds Ratios) of a multivariable logistic regression model with the lowest AIC found after backwards stepwise selection predicting a diagnosed SARS-CoV-2 infection between 01.01.2022 and 31.03.2022. The table includes Akaike Information Criterion (AIC), and Area Under the Curve (AUC). P values for each coefficient from the Wald test are denoted by ∗p<0.05* , ∗∗p<0.01, ∗∗∗p<0.001. The very high odds ratios for sequence category “I” is likely due to data separation between the sequence category and total number of vaccines predictor.
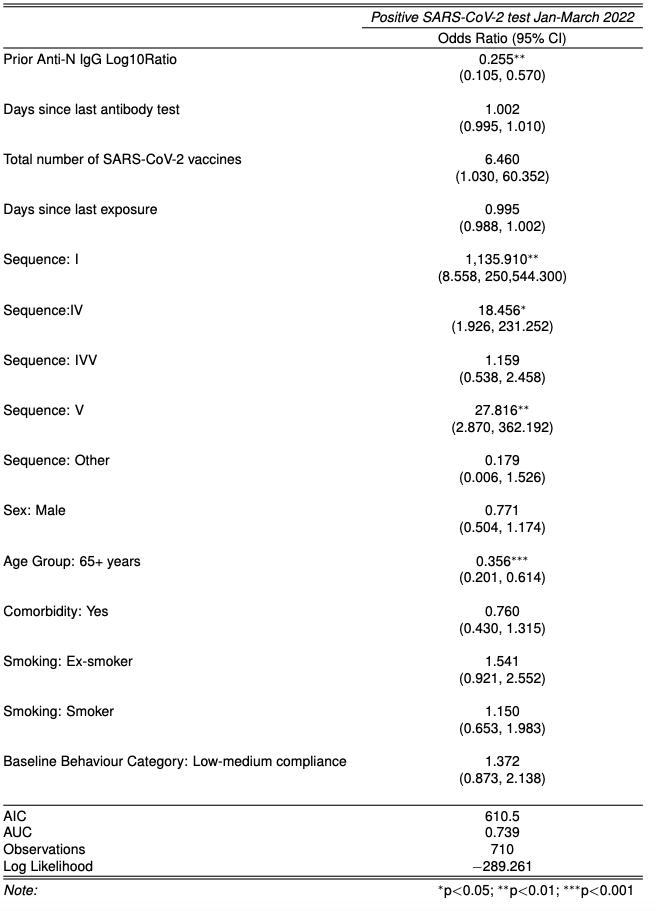


**Supplementary Table 6:** Output (Odds Ratios) of a multivariable logistic regression model with the lowest AIC found after backwards stepwise selection predicting a likely SARS-CoV-2 infection between 01.01.2022 and 30.06.2022. The table includes Akaike Information Criterion (AIC), and Area Under the Curve (AUC). P values for each coefficient from the Wald test are denoted by ∗p<0.05* , ∗∗p<0.01, ∗∗∗p<0.001


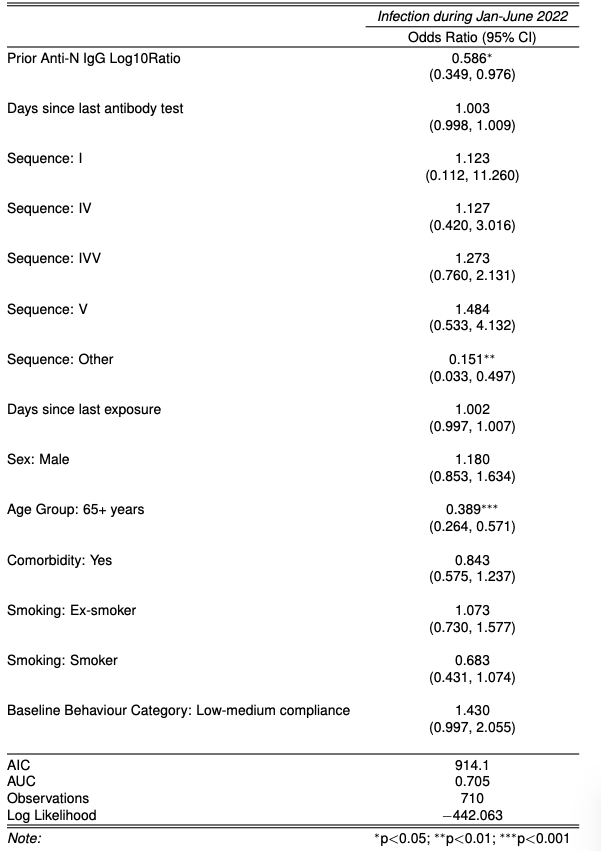

Supplement: Supplementary file 1 — Supplementary Material 1. [file 41512_2026_231_MOESM1_ESM.docx]
